# *Origanum vulgare* Terpenoids Induce Oxidative Stress and Reduce the Feeding Activity of *Spodoptera littoralis*

**DOI:** 10.3390/ijms19092805

**Published:** 2018-09-18

**Authors:** Chiara Agliassa, Massimo E. Maffei

**Affiliations:** Department Life Sciences and Systems Biology, University of Turin, Via G. Quarello 15/a, 10135 Turin, Italy; chiara.agliassa@unito.it

**Keywords:** *Origanum vulgare*, *Spodoptera littoralis*, terpenoid biosynthesis, larval survival, antioxidant enzyme activity and gene expression, toxicity

## Abstract

Terpenoids are toxic compounds produced by plants as a defense strategy against insect herbivores. We tested the effect of *Origanum vulgare* terpenoids on the generalist herbivore *Spodoptera littoralis* and the response of the plant to herbivory. Terpenoids were analyzed by GC-FID and GC-MS and quantitative gene expression (qPCR) was evaluated on selected plant genes involved in both terpene biosynthesis. The insect detoxification response to terpenes was evaluated by monitoring antioxidant enzymes activity and expression of insect genes involved in terpene detoxification. *O. vulgare* terpenoid biosynthesis and gene expression was modulated by *S. littoralis* feeding. The herbivore-induced increased level of terpenoids (particularly carvacrol and *p*-cymene) interacted with the herbivore by decreasing larval survival and growth rate. The assimilation by *S. littoralis* of more than 50% of ingested terpenes correlated with the possible toxic effects of *O. vulgare* terpenoids. In choice test experiments, carvacrol and γ-terpinene mediated the larval feeding preferences, wherease the prolonged feeding on *O. vulgare* terpenoids (particularly on γ-terpinene) exerted relevant antinutritional effects on larvae. *S. littoralis* was found to react to *O. vulgare* terpenoids by increasing its antioxidant enzymes activities and gene expression, although this was not sufficient to sustain the toxicity of *O. vulgare* terpenoids.

## 1. Introduction

Terpenoids constitute the largest and most heterogeneous class of secondary metabolites, and include monoterpenes and sesquiterpenes as volatile constituents [1,2]. These volatile terpenoids can act both as constitutive and herbivory-induced (Herbivore-Induced Plant Volatiles, HIPVs) defense compounds. Moreover, HIPVs can be emitted either at the site of damage or systemically from undamaged parts of affected plants [3]. Constitutive accumulation of terpenoids occurs in specialized tissues, such as the glandular trichomes [4], which are present in aromatic plants and act as direct defense against herbivores through either a toxic or deterrent activity [5]. In the *Lamiaceae* family, oregano (*Origanum vulgare* L.) is characterized by large peltate glandular trichomes that accumulate monoterpenes and sesquiterpenes [4,6,7]. The main terpenes are the two phenolic monoterpenes: Thymol and carvacrol [8,9,10]. These terpenoids are physiologically toxic towards insect larvae [11]. Phytophagous insects represent one of the major causes of biotic stress for plants [12] and herbivore attack can cause both quantitative and qualitative changes in the composition of HIPVs [2,13,14,15]. Monoterpenes such as thymol and carvacrol show LD_50_ values of 25 and 43 μg·larvae^−1^, respectively, when tested on early four-instar *Spodoptera litura* and the LD_50_ of these monoterpenes increases with the larval development [16]. The toxicity of these monoterpenes is higher versus *Spodoptera littoralis, Plutella xylostella*, *Callosobruchus maculatus* and *Culex quinquefasciatus*, with LD_50_ values as low as 0.22 μg·larvae^−1^ [17,18,19,20,21], whereas thymol and carvacrol toxicity versus *Sitophilus granarius* is lower, with LD_50_ above 100 μg·larvae^−1^ [22,23]. Toxic synergistic effects of thymol and carvacrol with other monoterpenes have been also described [24]. The mode of action of these monoterpenes on herbivores is not fully understood, although they have been found to elicit insect detoxification enzymes [21,25,26] and other mechanisms of tolerance [27].

The objective of this work was to study the response of the generalist herbivore *Spodoptera littoralis* to *Origanum vulgare* terpenoids. To this aim, we analyzed *O. vulgare* volatile terpenoids before and after herbivore feeding and evaluated their toxicity by insect exposure during feeding, while the terpenoid deterrent and anti-nutritional effects were tested by behavioral assays. Finally, the detoxification mechanisms of *S. littoralis* towards *O. vulgare* terpenes were also studied.

## 2. Results

### 2.1. O. vulgaris Terpenoids are Modulated by S. littoralis Herbivory

The volatile profile of *O. vulgare* undamaged leaves is characterized by the presence of several monoterpenes and sesquiterpenes (Table 1). In general, herbivory increased the total terpene content of *O. vulgare* leaves; however, the qualitative composition of terpenoids was not changed (Table 1). In particular, herbivore wounded (HW) *O. vulgare* plants showed a significant increase of two green leaf volatiles, (*E*)-2-hexenal and (*Z*)-3-hexenol, with respect to control undamaged plants. With regards to monoterpenes, HW significantly increased the content of α-terpinene, limonene, β-phellandrene, *trans*-sabinene hydrate, *cis*-sabinene hydrate, linalool, linalool oxide, α-terpineol, *trans*-dihydrocrvone, *cis*-dihydrocarvone, carvacrol methyl ether, linalyl acetate and thymol, with respect to controls. No significant difference was found for the remaining monoterpenes, including the major compound carvacrol (Table 1). Considering the sesquiterpenes, herbivory significantly increased the content of almost all identified compounds, with the exception of α-copaene, α-humulene and *trans*-farnesol (Table 1).

**Figure 1:**
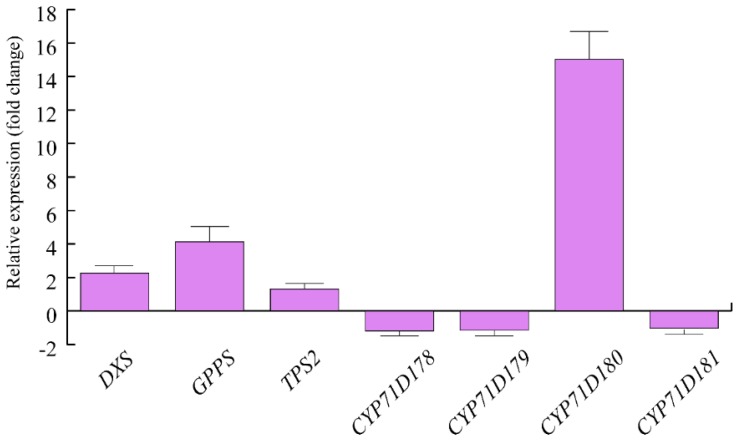
Levels of gene expression in *Origanum vulgare* leaves upon *Spodoptera littoralis* herbivory. Data are expressed as fold change (herbivory vs. control). *CYP71D180* = one of the 4 cytochrome P450 isoforms possibly involved in carvacrol biosythesis, isolated from *O. vulgare*; *TPS2*: γ-terpinene synthase, isolated from *O. vulgare* and involved in the production of several monoterpenes, including the potential precursors of carvacrol; *CYP71D178* and *CYP71D179* = cytochrome P450 isoforms possibly involved in carvacrol biosynthesis, isolated from *O. vulgare*; *DXS*: Deoxyxylulose phosphate synthase; *GPPS*: Geranyldiphosphate synthase. Three technical replicates were run for each biological replicate. Metric bars indicate standard deviation.

We then assessed the expression of some genes involved in *O. vulgare* terpenoid biosynthesis. Upon herbivory, a strong upregulation was found for *O. vulgare CYP71D180*, which was followed by a 4-fold upregulation of *GPPS* and an approximate 2-fold upregulation of *DXS*. *TPS2* showed minor upregulation after HW. *CYP71D178*, *CYP71D179* and *CYP1D181* were slightly downregulated by *S. littoralis* feeding activity (Figure 1).

### 2.2. O. vulgaris Terpenoids Exert Toxic Effects on S. littoralis larvae

**Figure 2:**
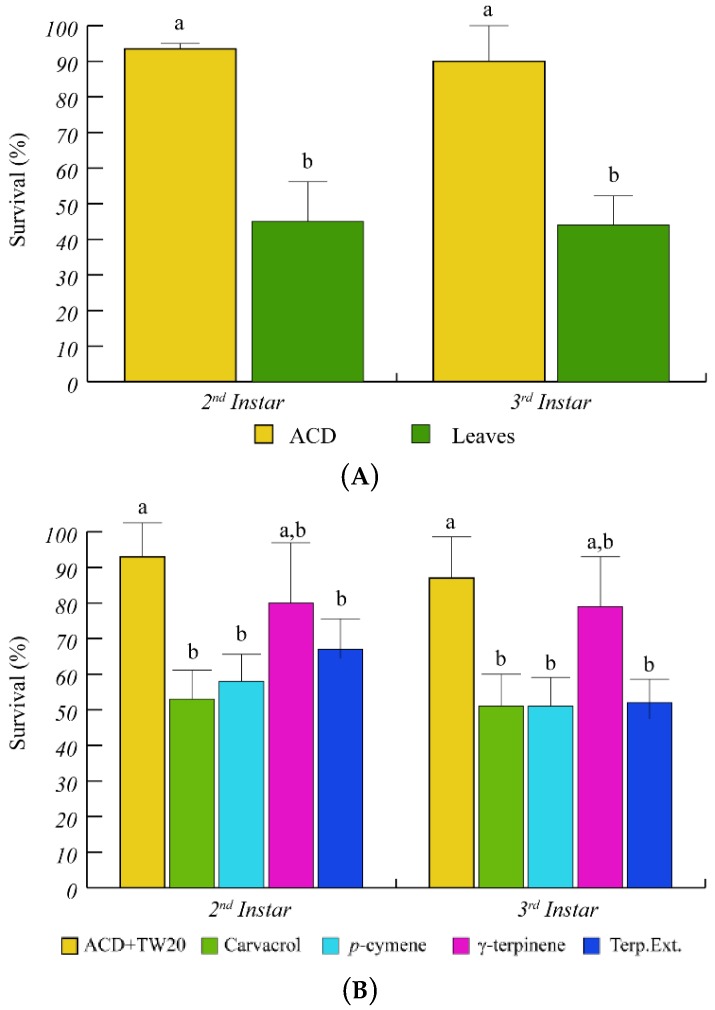
Percentage of *S. littoralis* survival upon feeding on *O. vulgare* leaves and terpenoids. (**A**) Comparison of *S. littoralis* survival upon feeding on artificial diet (ACD) or *O. vulgare* leaves. (**B**) Effect of terpenes extracted from *O. vulgare* leaves and of some *O. vulgare* monoterpenes on *S. littoralis* survival. ACD + Tween 20 was used as control. Bars indicate standard deviation. Different letters indicate significant (*p* < 0.05) differences.

Having assessed the ability of *O. vulgare* to respond to *S. littoralis* feeding by modulating terpenoid biosynthesis and gene expression, we tested the effect of *O. vulgare* terpenoids on *S. littoralis* larvae. After several trials we found that the 2nd and 3rd instar larvae showed a higher sensitivity to *O. vulgare* terpenoids; therefore, we used these two instar stages for all tests and measurements were performed during transition from one instar to the next one. Feeding on *O. vulgare* leaves caused a significantly (*p* < 0.05) lower survival percentage of *S. littoralis* larvae, with respect to the artificial diet (ACD) (Figure 2A). When larvae were offered a diet containing the *O. vulgare* terpenoid extract, a significant reduction of larvae survival was found with respect to ACD + Tween 20 (Figure 2B). We preliminary tested the effects of several *O. vulgare* terpenoids and we found that carvacrol (the major terpenoid of *O. vulgare*, Table 1) and *p*-cymene induced a significant (*p* < 0.05) reduction of survival percentage with respect to ACD + Tween 20, whereas the effect of γ-terpinene (the precursor of carvacrol) was not different from controls (Figure 2B).

**Figure 3:**
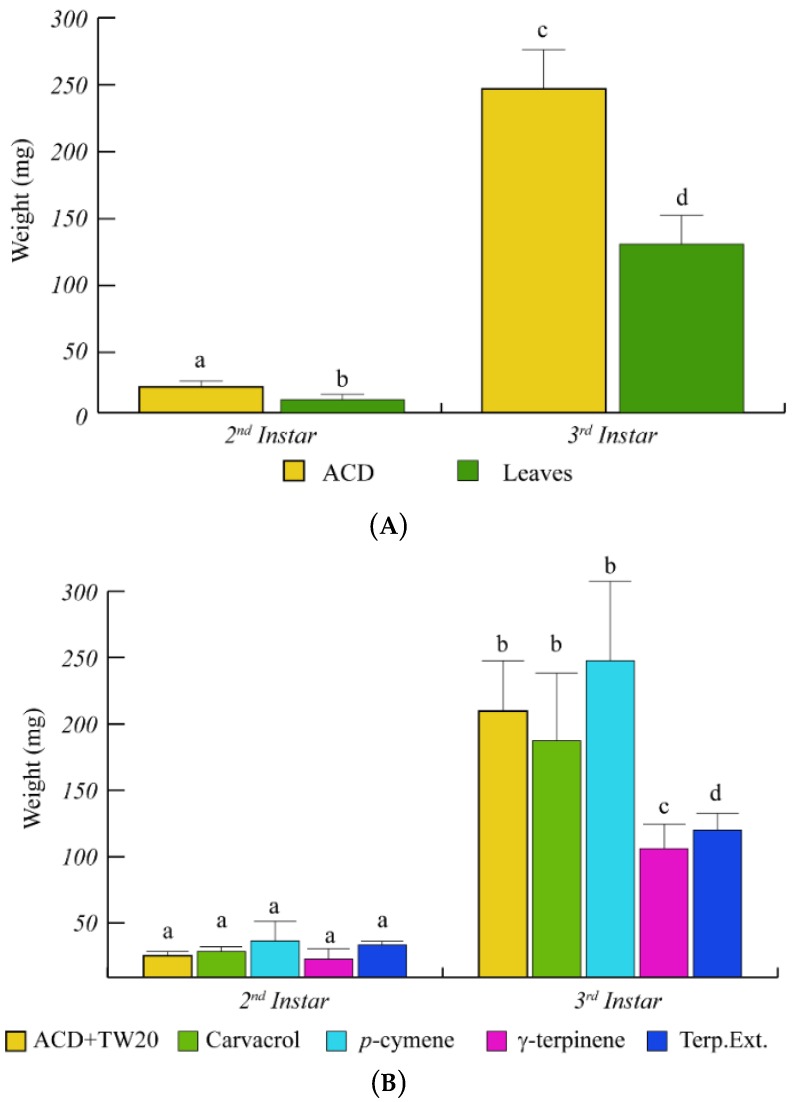
Weight variation of *S. littoralis* upon feeding on *O. vulgare* leaves and terpenoids. (**A)**. Comparison of *S. littoralis* weight upon feeding on ACD or *O. vulgare* leaves. (**B**). Effect of terpenes extracted from *O. vulgare* leaves and some monoterpenes produced by *O. vulgare* on *S. littoralis* weight. ACD + Tween 20 was used as control. Bars indicate standard deviation. Different letters indicate significant (*p* < 0.05) differences.

*O. vulgare* terpenoids also affected the larval weight with a significant (*p* < 0.05) weight reduction, with respect to ACD (Figure 3). In particular, during the larval development from the 2nd to the 3rd instar, the caterpillar weight increased less rapidly in caterpillars feeding on *O. vulgare* leaves, with respect to controls (Figure 3A). Caterpillars feeding on γ-terpinene and on *O. vulgare* terpenoid extracts showed a significant (*p* < 0.05) weight reduction with respect to ACD + Tween 20 (Figure 3B), whereas neither carvacrol nor *p*-cymene affected the larvae weight (Figure 3B). 

**Figure 4:**
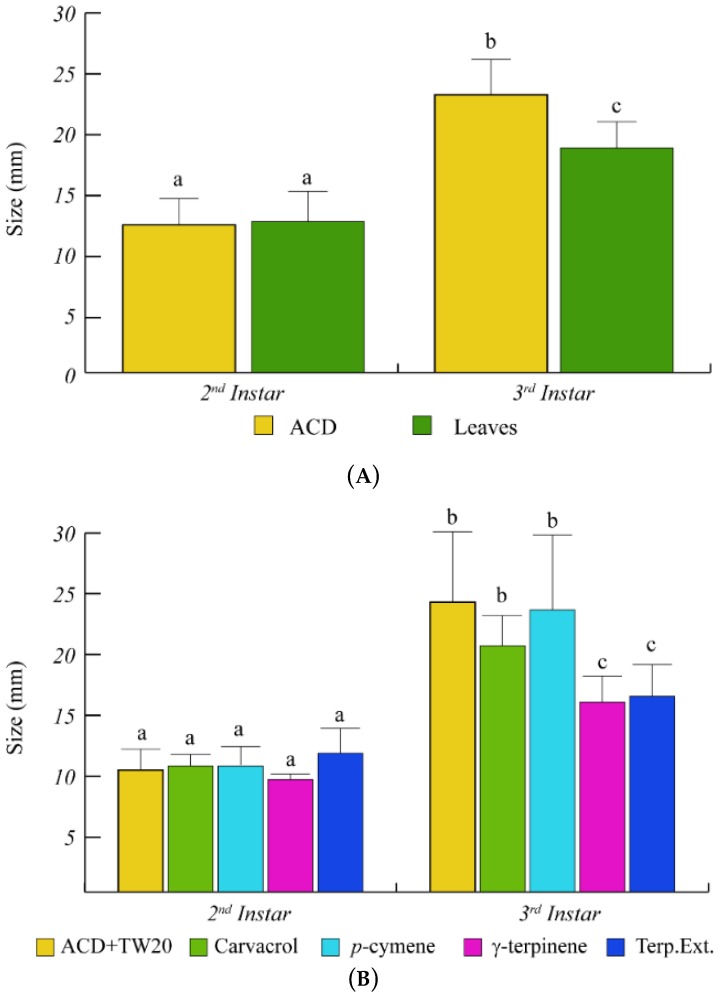
Larvae size of *S. littoralis* upon feeding on *O. vulgare* leaves and terpenoids. (**A**). Comparison of *S. littoralis* larvae size upon feeding on ACD or *O. vulgare* leaves. (**B**). Effect of terpenes extracted from *O. vulgare* leaves and some monoterpenes produced by *O. vulgare* on *S. littoralis* larvae size. ACD + Tween 20 was used as control. Bars indicate standard deviation. Different letters indicate significant (*p* < 0.05) differences.

To better assess the effects of *O. vulgare* terpenoids on *S. littoralis* growth, the increase of larval size was also monitored (Figure 4). Feeding of *S. littoralis* on *O. vulgare* leaves was ineffective on larvae size during the 2nd instar. However, a significant size reduction was found at the 3rd instar, with respect to controls (Figure 4A). As for the larvae weight increase, γ-terpinene and the *O. vulgare* terpenoids extracts were the only treatments that were able to significantly reduce the larvae size, with respect to ACD + Tween 20 (Figure 4B).

### 2.3. Priming of S. littoralis with O. vulgare Terpenoids Induces Different Choice Behaviors

**Figure 5:**
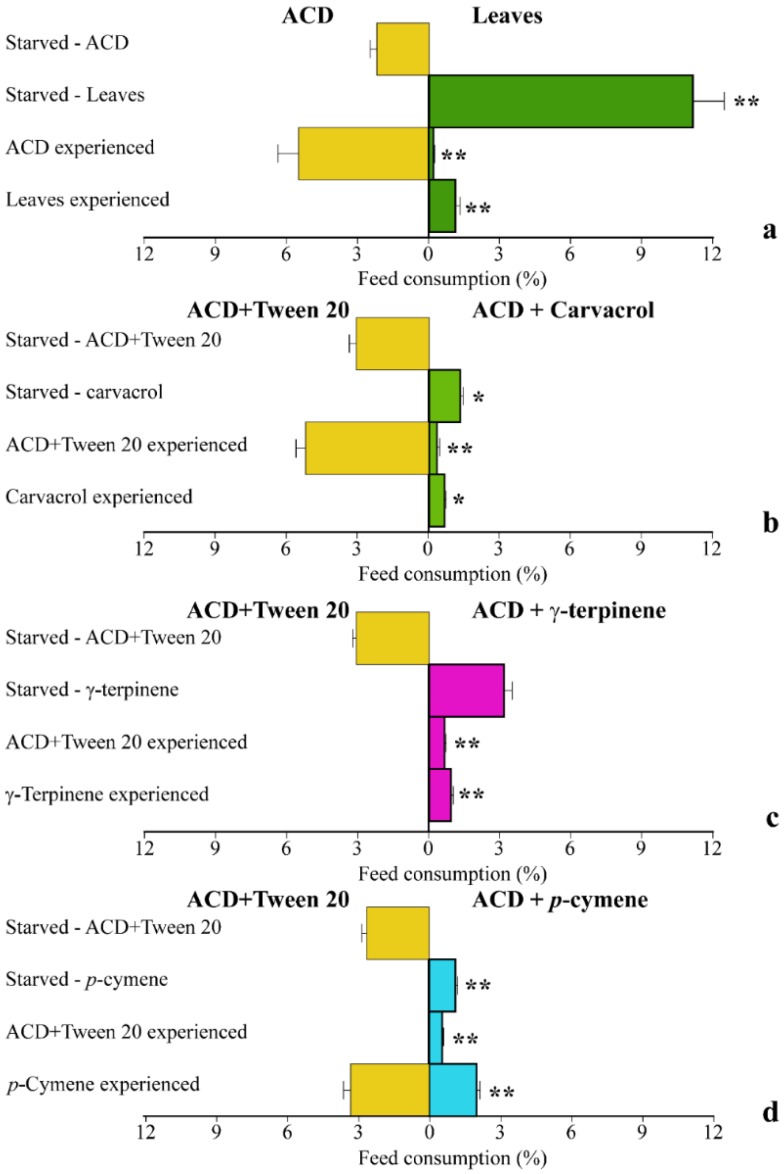
Behavior of *S. littoralis* upon feeding on *O. vulgare* leaves and terpenoids. (**a**). Response of *S. littoralis* starved larvae feeding on either ACD or *O. vulgaris* leaves along with feed preferences in larvae experiencing a previous nutrition with either ACD or *O. vulgare* leaves. (**b**). Response of *S. littoralis* starved larvae feeding on either ACD or ACD + carvacrol along with feed preferences in larvae experiencing a previous nutrition with either ACD or ACD + carvacrol. (**c**). Response of *S. littoralis* starved larvae feeding on either ACD or ACD + γ-terpinene along with feed preferences in larvae experiencing a previous nutrition with either ACD or ACD + γ-terpinene. (**d**). Response of *S. littoralis* starved larvae feeding on either ACD or ACD + *p*-cymene along with feed preferences in larvae experiencing a previous nutrition with either ACD or ACD + *p*-cymene. Results are expressed as the percentage of feed consumption. Values are the mean of at least three replicates; metric bars indicate standard deviation. Statistical difference is indicated by asterisks: * *p* < 0.05, ** *p* < 0.01.

We then compared the behavior of *S. littoralis* larvae with different feeding experience on *O. vulgare* leaves or ACD with *O. vulgare* terpenoids. In starved *S. littoralis* larvae, a significantly (*p* < 0.01) higher percentage of feed consumption was found when leaves were offered as the only feed source with respect to ACD (Figure 5a). However, when larvae were first fed with the ACD (ACD experienced), they significantly (*p* < 0.05) preferred to feed on ACD, with respect to leaves. However, larvae previously feeding on leaves (Leaf experienced) did not feed on ACD and consumed a significantly (*p* < 0.05) lower percentage of leaves, with respect to starved larvae (Figure 5a). We then evaluated the effect of the individual *O. vulgare* monoterpenes on larvae choice tests. Starved larvae significantly (*p* < 0.05) preferred to feed on ACD when offered as the only feed source with respect to ACD + carvacrol (Figure 5b) and the same results were found on ACD experienced larvae. However, carvacrol experienced larvae did not feed on ACD and significantly (*p* < 0.05) preferred to feed on ACD + carvacrol (Figure 5b). In the choice test using γ-terpinene, starved larvae did not show any significant difference in preference between ACD and ACD + γ-terpinene; however, both ACD experienced and γ-terpinene experienced larvae did not feed on ACD (Figure 5c). Finally, staved larvae preferentially fed on ACD with respect to ACD + *p*-cymene, whereas ACD experienced larvae fed preferentially on γ-terpinene. However, larvae experiencing γ-terpinene preferred to feed on ACD (Figure 5d).

### 2.4. S. littoralis Differentially Catabolizes O. vulgare Terpenoids

To evaluate the ability of *S. littoralis* to catabolize *O. vulgare* terpenoids, we analysed the terpenoid content of the frass of larvae (e.g., the insect excreted material) feeding on *O. vulgare* leaves (Table 1). The comparative analysis between leaf and frass terpenoids showed that many leaf *O. vulgare* volatiles were present in the insect frass and that the total terpenoid content was significantly reduced, indicating a high larval catabolic activity. In particular, the content of the major monoterpenes carvacrol, *cis*-sabinene hydrate, γ-terpinene and sabinene were significantly (*p* < 0.05) reduced in the frass, whereas most of the leaf monoterpenes and sesquiterpens were fully catabolized (Table 1).

### 2.5. O. vulgare Terpenoids Modulate the Activity and Expression of S. littoralis Scavenging and Detoxification Systems

**Figure 6:**
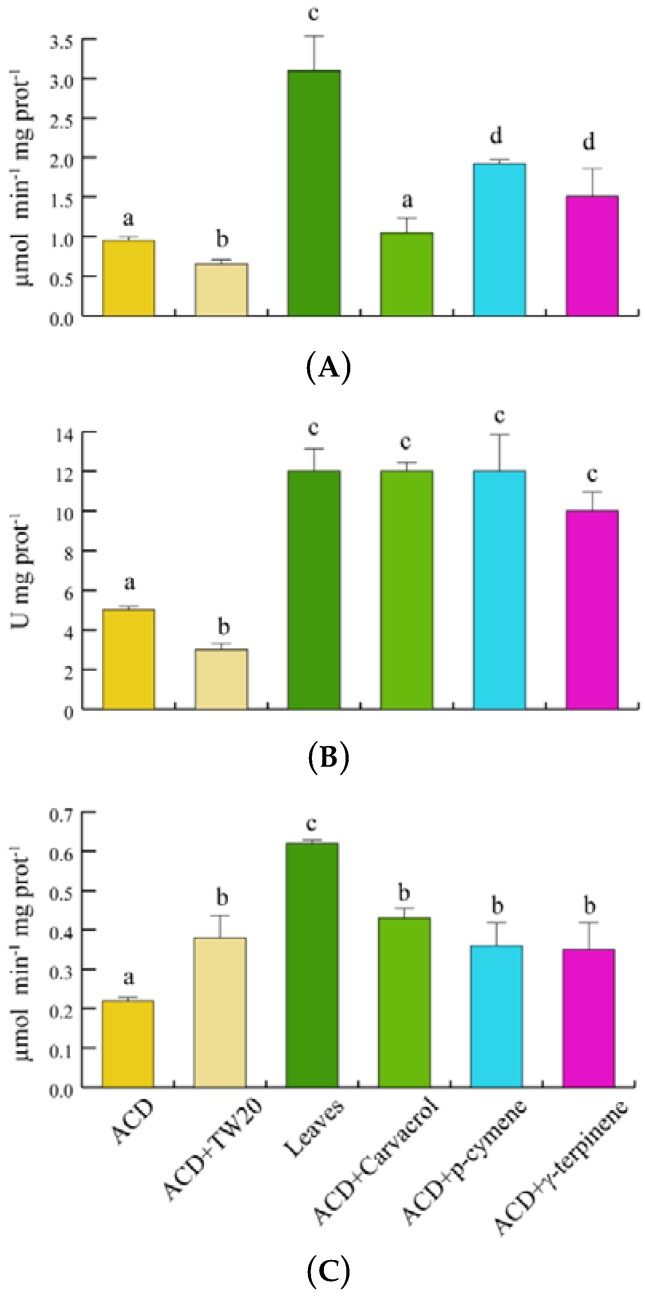
Antioxidant enzymes activities of *S. littoralis* after feeding on *O. vulgare* leaves and terpenoids. (**A**). Catalase (CAT) activity. (**B**). Superoxide dismutase (SOD) activity. (**C**). Glutathione *S*-transferase (GST) activity. Values are the mean of at least three replicates; metric bars indicate standard deviation. Different letters indicate significant (*p* < 0.05) differences.

In order to assess the potential scavenging responses of *S. littoralis* to *O. vulgare* terpenoids, we evaluated the insect’s catalase (CAT), superoxide dismutase (SOD) and glutathione *S*-transferase (GST) enzyme activities (Figure 6). With respect to ACD, feeding on *O. vulgare* leaves significantly (*p* < 0.05) increased the CAT activity, whereas a significant increase in CAT activity was found only for *p*-cymene and γ-terpinene, when compared to ACD + Tween 20 (Figure 6A). SOD activity significantly (*p* < 0.05) increased with respect to both ACD and ACD + Tween 20 when larvae fed on both *O. vulgare* leaves and *O. vulgare* terpenes (Figure 6B). Finally, GST activity was only significantly (*p* < 0.05) increased in larvae feeding on *O. vulgare* leaves, not being significantly affected by the individual terpenes (Figure 6C).

**Figure 7:**
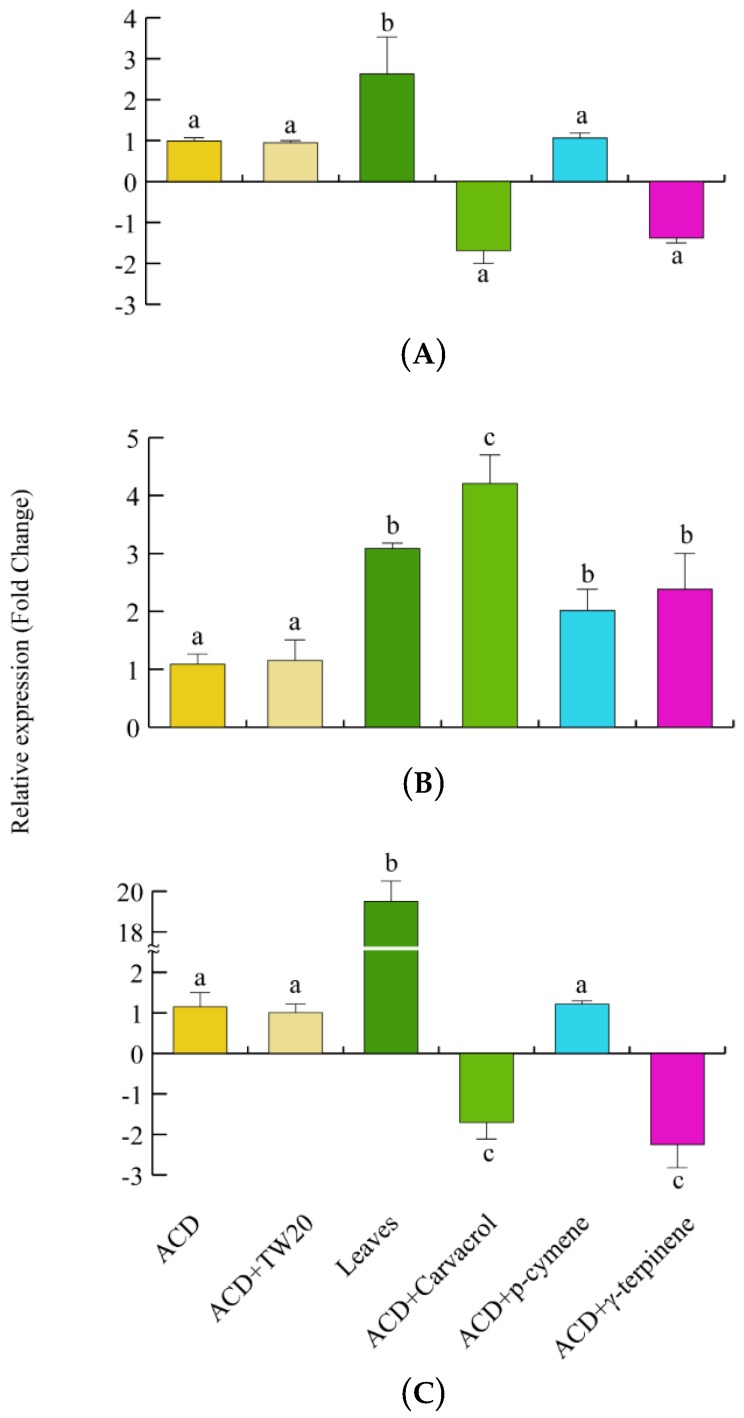
Levels of gene expression in *S. littoralis* upon feeding on *O. vulgare* with respect to those feeding on ACD or ACD + *O. vulgare* terpenoids (which is set to 1). (**A**). Catalase (*CAT*). (**B**). Superoxide dismutase (*SOD*). (**C**); Glutathione *S*-transferase (*GST*). Values are the mean of at least three replicates; metric bars indicate standard deviation. Different letters indicate significant (*p* < 0.05) differences.

We then assessed the gene expression of the herbivore scavenging and detoxifying genes. *CAT* expression was significantly upregulated when larvae fed on *O. vulgare* leaves, whereas no significant gene regulation was observed after feeding larvae with the individual terpenes (Figure 7A). *SOD* gene expression was always upregulated, particularly after carvacrol feeding, in larvae feeding on both *O. vulgare* leaves and terpenoids (Figure 7B). Finally, a significant and consistent upregulation of *GST* was found when larvae fed on oregano leaves, whereas carvacrol and γ-terpinene significantly (*p* < 0.05) downregulated the larvae *GST* expression (Figure 7C).

## 3. Discussion

Terpenoids stored in the secretory structures of aromatic plants act as direct defense against herbivores, particularly against generalist insects [13,28] and despite their constitutive nature, terpenoids can be modulated by herbivory [29,30,31,32]. In this work, we showed that feeding *S. littoralis* on *O. vulgare* leaves caused both the chemical and genetic modulation of the plant terpenoids. However, this modulation was mainly quantitative, because no qualitative changes were found in the terpenoids, with respect to control plants. In the interaction between the specialist *Chrisolina herbacea* and its host plant *Mentha acquatica*, terpenoid qualitative changes were observed upon herbivory [29]. Therefore, our results confirm the hypothesis that generalist and specialist herbivores may activate different plant responses [27]. The feeding activity of the generalist *S. littoralis* increased the expression of *O. vulgare DXS*, a gene involved in the early steps of terpenoid biosynthesis for the mevalonate-independent (MEP)-pathway gene, the product of which is considered to catalyze one of the rate-limiting steps of this pathway [33] as well as *GPPS*, whose overproduction is expected to result in increased production of monoterpene end products [34]. Upregulation of both *DXS* and *GPPS* correlated with the increased content of terpenoids upon herbivory. It is interesting to note that the specialist *C. herbacea* was unable to regulate the expression of *M. aquatica DXS* [29]. Of particular relevance was the herbivore-induced upregulation of γ-terpinene synthase (*TPS2*) and *CYP71D180*. CYP71D180 belongs to the cytochrome P450 (CYP) monooxygenases and is involved in further modifications of γ-terpinene backbone to yield carvacrol [8]. The gene is modulated by methyl jasmonate and salicylic acid as well as by feeding of insects [10] and its modulation is associated with a corresponding expression of early terpenoid genes, like *DXP* [35]. *TPS2* increased expression has been recently reported upon interaction of *O. vulgare* with a *Myrmica* ant [10] and confirms the modulation of this gene upon biotic attack. Besides terpenoids, qualitative differences were found in the production of some GLVs, which are considered typical wound-related VOCs [13,36,37,38,39]. Upon herbivory, a significant release of GLVs has been observed in several non-aromatic plants, such as lima bean [40], as well as in plants producing glandular trichomes like *Monarda fistulosa* [41] and tomato [39].

The toxicity of terpenoids toward herbivory has been widely documented [42] and the insecticidal effect of several monoterpenes has been demonstrated in many species belonging to the Lamiaceae family [43,44,45,46]. Several studies have evaluated the toxicity of plant terpenes by the use of essential oils. However, the essential oil does not reflect the natural composition of accumulated terpenes in the glandular trichomes since partial terpene recovery and thermal degradation can occur [47]. In our study we used intact leaves and terpenes extracted from *O. vulgare* leaves to perform toxicological tests and we exposed the caterpillars to physiological concentrations based on the quantity detected in *O. vulgare* leaves. Carvacrol and *p*-cymene were the most toxic terpenes among the tested monoterpenes. These results are in agreement with the reported toxicity of carvacrol [48]. In a study on the toxicity of carvacrol and *p*-cymene towards *S. littoralis*, carvacrol resulted more active (LD_25_ = 7 µg·larva^−1^) with respect to *p*-cymene (LD_25_ = 25 µg·larva^−1^) [17]. We also observed that the survival percentage of larvae upon carvacrol and *p*-cymene feeding was higher than larvae fed on leaves at the end of 3rd stage. Toxicology studies on *S. littoralis* showed that the binary mixture of carvacrol and *p*-cymene have a synergic toxic effect if compared with the single compounds [17]. Therefore, similar synergistic effects can explain the lower toxicity of individual compounds with respect to *O. vulgare* leaves. Despite the known effects of thymol as an insecticide [49,50,51], no significant effects were observed in our study, probably because of the very low amount of thymol produced by the *O. vulgare* chemotype used in this study.

The inhibition of larval growth was mainly due to the presence of γ-terpinene, the biosynthetic precursor of carvacrol [8,52], whereas carvacrol and *p*-cymene did not affect significantly the larval growth. Although the topical application of a sub-lethal concentrations of carvacrol to *S. littoralis* has been demonstrated to exert a significant delay of larval development [16], feeding on carvacrol was found to be more tolerated by the insect, possibly by a different biodegradation/detoxification of this compound in the insect’s gut. Since the growth rate, development lifespan, final body weight and survival percentages are strongly affected by the food intake and by the nutritional value of ingested food [53], it is conceivable that the larvae growth delay and reduced survival observed upon feeding on *O. vulgare* leaves might be correlated to both the deterrent and anti-nutritional effect of oregano terpenoids. Although the naïve larvae preferred the ACD during the choice tests, the presence of leaves as alternative food source appears to stimulate a higher feeding rate. In fact, larvae that experienced feeding on leaves selectively preferred *O. vulgare* leaves as nutritive source during the choice test. Our results also indicate a possible attractant effect mainly mediated by carvacrol and γ-terpinene [54,55]. Our data suggest that the continuous feeding on *O. vulgare* leaves significantly impairs the larvae growth (weight and size) and that this anti-nutritional effect is mainly mediated by γ-terpinene. 

*S. litura* larvae are able to detoxify terpenes, including γ-terpinene [56,57,58]. The absence of chemical derivatives of *O. vulgare* terpenes and the assimilation of more than 50% of ingested terpenes by *S. littoralis*, as evidenced by our insect’s frass analyses, show that *S. littoralis* catabolizes the plant terpenes and shows a limited ability to biotransform these molecules, in contrast to the typical behavior of specialist herbivores [59]. However, the catabolism of *O. vulgare* terpenes can also produce non-volatile derivatives which might have not been detected by our GC-MS and GC-FID analyses. Despite the absence of catabolic products, the insect response to ingestion of *O. vulgare* leaves was a significant increase of all tested enzyme activities. This increase was correlated to the presence of some *O. vulgare* monoterpenes only for CAT and SOD, whereas the increased GST activity was probably dependent on other leaf constituents or by the synergistic effect of the plant terpenoid blend. Increased oxidative stress is common in herbivore insects feeding on plants producing toxic allelochemicals. Insects like *S. littoralis* possess a suite of antioxidant enzymes such as CAT and SOD for protection against oxidative stress [60]. Insects also possess a GST which is effective in targeting hydroperoxides [61] and some terpenoid have the ability to modulate the activity of this enzyme [21]. For all tested enzymes and genes, there was a positive correlation between insect enzyme activity and gene expression upon feeding on leaves. In *S. littoralis*, a significant up-regulation of *SOD*, *CAT* and *GST* was shown upon feeding on a diet containing potato extracts showing that increased concentrations of antioxidants represent an herbivore defense against exogenous oxidative stress [62]. In agreement with the general hypothesis that increased oxidative stress may lead to a modulation of genes coding for antioxidant enzymes [62], we also found a positive correlation between SOD and CAT activity and gene expression upon feeding on selected monoterpenes. The enhanced activity of insect’s SOD and CAT prevents oxidative damage and could depend on its secretion into the gut [62,63]. GST enzyme activity was not modulated by the selected terpenoids while its gene expression was downregulated by carvacrol and γ-terpinene. It is possible that the observed toxic effect of these monoterpenes may be partly depending on the reduced expression of *GST*. However, the contrasting results between the modulation exerted by feeding on leaves does not exclude the possibility that the regulation of this genes could be associated to other *O. vulgare* constituents such as phenolics [64,65,66]. It is known that some surfactants may interfere with enzyme activities [67]. Interestingly, the use of Tween-20 (which was essential for terpenoid solubilization) affected the enzyme activities of all tested enzymes.

## 4. Materials and Methods 

### 4.1. Plant and Animal Material

*Origanum vulgare* L. (Lamiaceae) plants were propagated from stem cuttings provided by the University of Turin (Italy) Botanical Garden and grown with fluorescent lamps (200 µmol·m^−2^·s^−1^) with a light/darkness photoperiod of 16/8 h, 60% humidity at a temperature of 22 °C ± 2 °C. *Spodoptera littoralis* Boisd. (Lepidoptera, Noctuidae) were kindly supplied as egg clutches by Syngenta Crop, Protection Münchwilen AG (Stein, Switzerland). Larvae were reared in plastic Petri dishes with the artificial diets as specified below, at 25 °C, 60% relative humidity and a light/darkness photoperiod of 16/8 h.

### 4.2. Chemicals Used

Carvacrol (95%), *p*-cymene (95%), thymol (95%) and γ-terpinene (95%), were purchased from TCI-EUROPE (Belgium). The chemical standards were properly diluted in water with the addition of 0.1% *w*/*v* Tween 20 (polyoxy ethylene sorbitan monolaurate, Sigma-Aldrich, Milan, Italy).

### 4.3. Extraction of O. vulgare Terpenoids

*O. vulgare* terpenoids were extracted from 20 g leaves with 70% ethanol (Sigma-Aldrich, USA) with a 1:40 *w*/*v* ratio. Extraction was performed overnight in the dark at 22 °C and then in a sonic bath for 30 min at 22 °C. The extract was filtered with cheese cloth and the leaves were re-extracted in a mortar with 70% ethanol. The extract was then filtered and both filtrates were combined and centrifuged for 10 min at 4000× *g* to remove plant residues. Terpenoids were then separated by liquid/liquid extraction (1:1 *v*/*v*) in a separation funnel with a mixture of 4:1 *v*/*v* hexane:pentane (Carlo Erba, Milan, Italy). The liquid/liquid extraction was repeated twice. The extract was concentrated under vacuum by Centrivap concentrator (Labconco, Kansas City, MO, USA) at 35 °C and then reduced to 3 mL by a constant flow of nitrogen.

### 4.4. Artificial Diet Composition

The artificial control diet (ACD) was composed of 125 g dry kidney beans, 2.25 g ascorbic acid (Sigma-Aldrich, Milan, Italy), 2.25 g ethyl hydroxybenzoate (Fluka, Milan, Italy), 750 µL formaldehyde (Fluka), 10 g plant agar (Duchefa, Haarlem, The Netherland) and 600 mL water. The artificial diet was stored at −20 °C until use. The ACD was supplemented with *O. vulgare* extracted terpenes, carvacrol, *p*-cymene and γ-terpinene with the addition of 0.1% *w*/*v* Tween 20. To avoid thermal degradation and volatilization of terpenes, the ACD was cooled down to 40 °C before the addition of the aforementioned terpenes and terpenoid extract. Terpenes were added to the diet in quantities comparable to those detected in the leaves eaten by the caterpillars (i.e., the amounts described in Table 1).

### 4.5. Terpene Toxicity Assays

Toxicological experiments were carried out from the 2nd instar until the end of the 3rd instar of *S. littoralis* larvae reared on the ACD. Each caterpillar was moved into a new Petri dish and daily fed with either *O. vulgare* leaves (by using ACD as a control) or ACD supplemented with the terpenes (by using ACT + Tween 20 as a control). The daily supply of diet or leaves was calculated on larvae instars (80 and 150 mg for the 2nd and 3rd instar, respectively). For each biological test, 15 biological replicates were run. During treatments, the survival percentage, larvae size and larvae weight were daily monitored. Larvae length was calculated with the use of ImageJ image software (NIH, Bethesda, ND, USA). 

### 4.6. Leaf Disk Choice Tests

Choice tests were conducted using 3rd instar larvae starved for 17 h before tests. The assay time was 6 h and for each test 15 biological replicates were run. Tests were carried out in square plastic Petri dishes (12 cm × 12 cm) where *O. vulgare* leaf disks or ACD plus terpenes were alternated with ACD pieces of similar area (1.7 cm^2^) on a 10 × 10 cm grid (see Supplementary Figure S1). At the beginning of the test, one *S. littoralis* larva was placed in the center of the grid. The Petri dishes were covered with black mosquito net to reduce the light exposure as suggested by Carroll et al. [68]. We tested either naïve larvae that were fed only with ACD until bioassays or experienced larvae which were exposed, the previous day, to *O. vulgare* leaves or ACD plus terpenes. At the end of each bioassay, the percentage of eaten feed was calculated and the weight loss due to water evaporation was assessed in order to normalize the results of feeding preferences.

### 4.7. S. littoralis Feeding on O. vulgaris Leaves

Overnight starved 3rd instar *S. littoralis* larvae were placed on *O. vulgare* branches. After 24 h, the caterpillars were removed and the herbivore damaged leaves were immediately collected and stored at −20 °C until extraction. Undamaged *O. vulgare* leaves were used as control.

150 mg herbivore-wounded (HW) *O. vulgare* leaves and controls were extracted in a glass tube with 3 mL 2:1 hexane/diethyl ether (Carlo Erba, Milan, Italy). Twenty µg pulegone (TCI Europe N.V., Zwijndrecht, Belgium) were added as internal standard.

*S. littoralis* frass was collected from 3rd instar caterpillars feeding on *O. vulgare* leaves. Samples were collected, weighted and stored at −20 °C until use. The experiment was performed in triplicate. One hundred mg frass were extracted with a pestle in a glass tube with 3 mL of 2:1 hexane/diethyl ether by using 20 µg pulegone as internal standard. The frass extract was then placed in an ultrasonic water bath at room temperature for 30 min and then centrifuged at 4000× *g* for 5 min and the supernatant dehydrated in a glass column packed with anhydrous MgSO_4_ (Fluka, Milan, Italy). The extract was then concentrated by a constant flow of nitrogen (N_2_) to 250 µL before GC-MS and GC-FID analysis. 

### 4.8. Qualitative and Quantitative Analyses of O. vulgare Leaves and S. littoralis Frass Terpenoids

Qualitative and quantitative analyses of volatile compounds in *O. vulgare* leaves and caterpillar frass were performed by GC-MS and GC-FID, respectively. GC-MS was performed with an Agilent 6890 N gas chromatograph coupled to an Agilent 5973A mass spectrometer by using a ZEBRON ZB-WAX column (30 m length, 250 µm diameter, 0.25 µm thickness) (Phenomenex, Torrance, CA, USA). Helium was used as carrier gas at constant flow of 1 mL·min^−1^. The following temperature program was used: 50 °C as initial temperature, thermal gradient of 2 °C min^−1^ up to 190 °C and then at 15 °C min^−1^ up to 250 °C. Post time lasted 2 min at 250 °C. Injector port was set at 250 °C in splitless mode. Transfer line temperature to MSD was 280 °C and ionization energy (EI) was 70 eV. Mass spectra were acquired in full scan mode with a 50–350 *m*/*z* range. The identification of compounds was based on the comparison of their mass spectra with NIST 98 by the NIST v2.0 research software (Standard Reference Data, Gaithersburg, MD, USA) comparison of retention indices from apposite literature papers [69,70] and pure standards. GC-FID quantitative analyses were performed on an Agilent 6890N gas chromatograph coupled to an FID at the same conditions as described above.

### 4.9. Isolation of Total RNA and Expression of O. vulgare Genes in Response to S. littoralis Herbivory

To evaluate the effect of *S. littoralis* herbivory on *O. vulgare* terpenoid metabolism, we analyzed the expression level of terpene synthases and cytochrome P450s previously known to be involved in the production of the main *O. vulgare* terpenoids [8,10,35]. Total RNA was extracted from 50 mg of *O. vulgare* leaves after 6 h *S. littoralis* feeding and control unwounded leaves using the Agilent Plant RNA Isolation Mini Kit (Agilent Technologies, Santa Clara, CA, USA). To remove residual genomic DNA, RNA was treated with RNAse-free DNAse I set (Qiagen, Venio, The Netherlands). RNA quality was checked using the Agilent 2100 Bioanalyzer with RNA 6000 Nano LabChip. Quantitative analysis was performed using the Nano Drop ND-1000 micro-scale spectrophotometer (Thermo Fisher Scientific, Wilmington, DE, USA). For cDNA synthesis, high-capacity cDNA Reverse Transcription Kit (Applied Biosystems, Foster City, CA, USA) was used according to manufacturer’s instructions. Reactions were prepared by adding 1.5 µg total RNA, 2 µL of 10× RT Buffer, 0.8 µL of 25× dNTPs mix (100 mM), 2 µL 10× RT primer, 1 µL of Multiscribe™ Reverse Transcriptase and nuclease-free sterile water up to 20 µL. Reaction mixtures were incubated at 25 °C for 10 min, 37 °C for 2 h, and 85 °C for 5 s. Samples were stored at −20 °C for further analyses. Primer pairs for the selected genes were designed using BeaconDesigner (version 5.0; PremierBiosoft, Palo Alto, CA, USA). Primers were designed for regions with the largest possible difference between the P450 sequences whilst primer binding sites were chosen for identical regions of all known alleles for the respective genes from *O. vulgare* and *Thymus vulgaris*. Each primer pair was tested for potential cross-hybridisation with the other P450s as templates; none was observed at a 60 °C annealing temperature except that the CYP71D179 primers cross-reacted to some extent with the CYP79D178 and D182 sequences due to their close sequence similarities. Primers are listed in Supplementary Table S1. Primer efficiencies for all pairs were calculated using the standard curve method [71]. 

qPCR analysis was on a Stratagene Mx3000P Real-Time System (La Jolla, CA, USA) using SYBR green I with ROX as internal loading standard, using 10 μL of mixture consisting of 5 µL 2X MaximaTM SYBR Green qPCR Master Mix (Fermentas, Waltham, MA, USA), 0.6 µL cDNA and 300 nM primers (Integrated DNA Technologies, Skokie, IL, USA). Controls included non-RT controls (using total RNA without reverse transcription to monitor genomic DNA contamination) and non-template controls (water template). PCR conditions were: 10 min at 95 °C, 40 cycles of 15 s at 95 °C, 20 s at 60 °C, 30 s at 72 °C. Fluorescence was read after each annealing and extension phase. All runs were followed by a melting curve analysis from 55 to 95 °C. The linear range of template concentration to threshold cycle value (*C*_t_ value) was determined by performing a dilution series using cDNA from three independent RNA extractions analyzed in three technical replicates. qPCR reactions were run using specific primers [52]. Three distinct reference genes: Elongation factor 1 alpha, actin and 18S rRNA were used to normalize the results of the real-time PCR. The most stable gene, selected using Normfinder software [72], was the elongation factor 1 alpha. All amplification plots were analyzed with MX3000P™ software (Agilent Technologies, Santa Clara, CA, USA) to obtain *Ct* values. Relative RNA levels were calibrated and normalized with the level of the elongation factor 1 alpha mRNA.

### 4.10. Isolation of Total RNA and Expression of S. littoralis Scavenging and Detoxifying Genes in Response to O. vulgare Terpenoids

Larvae were fed with ACD, ACD + Tween 20, ACD + *O. vulgare* terpenoids and *O. vulgare* leaves and were collected during the 3rd instar.

Frozen larvae total RNA was isolated using Qiagen RNeasy Micro kit and RNase-Free DNase set to remove residual genomic DNA. Sample quality and quantity was checked by using the RNA 6000 Nano kit and Agilent 2100 Bioanalyzer and confirmed spectrophotometrically using a NanoDrop ND-1000. High-capacity cDNA Reverse Transcription Kit was used for cDNA synthesis. Reactions were prepared by adding 500 ng total RNA and following the same protocol as described above. All qPCR experiments were performed on a Stratagene Mx3000P Real-Time System using SYBR green I with ROX as an internal loading standard. The reactions mixtures were prepared as above. PCR conditions were: Elongation factor 1 (*EF1*), actin (*ACT*), catalase (*CAT*), superoxide dismutase (*SOD*) and glutathione-*S*-transferase (*GST*): 10 min at 95 °C, 45 cycles of 15 s at 95 °C, 40 s at 52 °C, and 30 s at 72 °C, 1 min at 95 °C, 30 s at 55 °C, 30 s at 95 °C. Procedures for fluorescence reading, melting curve analysis and determination of the linear range of template concentration to *Ct* value were as described above. 

qPCR reactions were run using specific primers designed on Genbank-available *S. litura* and *S. exigua* sequences using the Primer3 software [73] (Supplementary Table S2), with efficiencies for all pairs calculated as above. Two reference genes, *ACT* and *EF1*, were used to normalize results of the real time PCR, from which *ACT* was selected as described above. *Ct* values of amplification plots and the calibration and normalization of relative RNA levels (using *ACT*) were as described above.

### 4.11. S. littoralis Scavenging and Detoxifying Enzyme Activities upon Feeding on O. vulgare Terpenoids

*S. littoralis* larvae at the transition between the 2nd and the 3rd instar were collected after 7 days feeding on the required diet and were immediately homogenized in 1 mL ice-cold 50 mM potassium phosphate pH 7.2 containing 0.5 mM EDTA and 10 mmol protease inhibitor phenyl-methyl-sulfonyl fluoride according to Hermeslima et al. [74]. The homogenate was centrifuged at 1600× *g* for 30 min at 4 °C, and the supernatant was stored at −20 °C for enzyme assays.

Catalase (EC 1.11.1.6) (CAT) activity was measured according to Vecera et al. [75] with a few modifications. Ten microliters of sample homogenate were mixed with 390 μL 60 mM K_2_PO_4_ buffer (pH 7.0) and poured into the glass cuvette. Then 400 μL 21 mM H_2_O_2_ solution were added and mixed, and the decrease in absorbance at 240 nm was measured. CAT activity was expressed in μmol of decomposed H_2_O_2_ per minute using the extinction coefficient of 39.4 M^−1^·cm^−1^.

Superoxide dismutase (EC 1.15.1.1) (SOD) activity was assayed according to Karthi and Shivakumar [76] with some modifications. The reaction mixture was prepared in 1 mL spectrophotometer cuvettes by using 50 mM Tris-HCl and 10 mM EDTA buffer (pH 8.2) and 15 μL extract supernatant. The content was mixed and the final volume was adjusted 990 μL. The reaction was started with the addition of 10 μL 30 mM pyrogallol and the absorbance read at 440 nm. One unit of total SOD activity was calculated as the amount of protein per milligram causing 50% inhibition of pyrogallol autoxidation. The total SOD activity was expressed as units per milligram of protein.

Glutathione *S*-transferase (EC 2.5.1.18) (GST) activity was assayed according to Habig et al. [77] with some modifications. The reaction mixture was prepared in 1 mL spectrophotometer cuvettes by using 50 mM Tris-HCl buffer (pH 7.5), 100 μL 0.4 mM 1-Chloro-2.4-dinitrobenzene and 100 μL extract supernatant. The reaction was started with the addition of 100 μL 4 mM reduced glutathione and the absorbance read at 340 nm. The GST activity was expressed as units per milligram of protein

### 4.12. Soluble Protein Determination

Soluble protein concentration was evaluated by the method of Bradford [78] using BSA as a standard.

### 4.13. Statistical Analyses

The proportion of survived larvae were statistically compared using a *z* test. The overall data sets are expressed as mean values of at least three biological replicates, using metric bars to indicate SD. Significance of differences observed in data sets was tested by ANOVA and then by the non-parametric Bonferroni and Tukey *post-hoc* test using the software SYSTAT 10 (SPSS Inc., Hong Kong, China).

## 5. Conclusions

In conclusion, the data here reported showed an increased modulation of *O. vulgare* terpenoid biosynthesis and gene expression upon *S. littoralis* feeding. The herbivore-induced increased level of terpenoids (particularly carvacrol and *p*-cymene) interacts with the herbivore by decreasing larval survival and growth rate. The assimilation by *S. littoralis* of more than 50% of ingested terpenes correlates with the possible toxic effects of *O. vulgare* terpenoids. Carvacrol and γ-terpinene mediate the larval feeding preferences; however, the prolonged feeding on *O. vulgare* terpenoids (particularly on γ-terpinene) exerts relevant toxic effects on larval survival. The insect reacts to *O. vulgare* terpenoids by modulating its antioxidant enzymes activities and gene expression; however, this was found insufficient to sustain the *O. vulgare* terpenoid toxicity.

## Figures and Tables

**Table 1:** Chemical composition of *Origanum vulgare* terpenoids in undamaged control plants, in *Spodoptera littoralis* herbivore wounded (HW) plants and in *S. littoralis* frass upon feeding on *O. vulgare* leaves. The results are expressed as µg g^−1^ fresh weight and are the mean of at least three replicates ± SD. Asterisks indicate significant differences between control and HW: * *p* < 0.05; ** *p* < 0.01.

Compounds	Control	HW	Frass
**Green Leaf Volatiles**			
(*E*)-2-Hexenal	nd	8.96 ± 3.66 **	nd
(*Z*)-3-Hexenol	nd	9.43 ± 0.79 **	nd
**Monoterpenes**			
Sabinene	116.32 ± 0.24	133.99 ± 8.09	2.84 ± 0.34
β-Pinene	4.18 ± 0.03	4.75 ± 0.56	nd
β-Myrcene	31.93 ± 1.00	34.61 ± 1.88	0.42 ± 0.04
α-Terpinene	25.62 ± 0.5	32.38 ± 2.30 *	0.77 ± 0.12
*p*-Cymene	53.87 ± 18.84	83.43 ± 10.39	2.97 ± 0.60
1-Octen-3-ol	5.12 ± 0.31	13.75 ± 3.67	nd
Limonene	14.55 ± 2.13	20.21 ± 0.05 *	nd
β-Phellandrene	17.91 ± 1.36	28.83 ± 2.95 *	nd
γ-Terpinene	187.89 ± 1.61	193.92 ± 15.28	8.60 ± 1.17
1-Octenyl-3-acetate	20.01 ± 0.93	24.75 ± 4.29	nd
*Trans*-sabinene hydrate	52.28 ± 5.31	98.07 ± 10.80 **	1.41 ± 0.32
*Cis-*sabinene hydrate	700.21 ± 71.55	1062.03 ± 167.15 **	19.38 ± 1.53
Linalool	7.40±0.49	17.79 ± 0.62 *	nd
Linalool oxide	4.33 ± 0.15	6.90 ± 0.74	nd
Terpinen-4-ol	8.12 ± 0.86	10.78 ± 2.03	1.16 ± 0.16
α-Terpineol	43.64 ± 4.56	60.50 ± 11.09 *	1.80 ± 0.20
*Trans-*dihydrocarvone	4.04 ± 0.28	5.88 ± 0.23 **	nd
*Cis*-dihydrocarvone	9.20 ± 0.56	12.70 ± 1.68 *	1.57 ± 0.29
Sabinene hydrate acetate	5.59 ± 0.09	6.76 ± 1.11	nd
Carvacrol methyl ether	44.29 ± 1.77	86.34 ± 5.03 **	3.35 ± 0.44
Linalyl acetate	51.03 ± 3.11	88.85 ± 10.51 **	2.45 ± 0.18
Thymol	4.11 ± 0.33	6.92 ± 0.32 **	nd
Carvacrol	959.26 ± 8.90	1159.39 ± 84.46	109.76 ± 6.96
**Sesquiterpenes**			
Bicycloelemene	11.49 ± 1.54	18.95 ± 1.32 *	nd
β-Cubebene	5.99 ± 0.92	12.13 ± 0.03 **	nd
α-Copaene	10.93 ± 1.31	7.73 ± 2.10	nd
β-Caryophyllene	40.81 ± 2.66	58.38 ± 5.43 *	1.69 ± 0.29
α-Humulene	5.65 ± 1.04	7.65 ± 0.20	nd
Germacrene D	37.19 ± 2.72	49.46 ± 2.96 *	1.79 ± 0.37
Bicyclogermacrene	24.05 ± 3.32	33.18 ± 2.12*	1.13 ± 0.12
β-Bisabolene	93.00 ± 7.33	114.85 ± 4.94 *	5.33 ± 0.93
α-Farnesene	2.54 ± 0.16	6.44 ± 0.59 *	nd
β-Sesquiphellandrene	6.91 ± 0.92	12.71 ± 0.80 **	nd
Germacrene d-4-ol	4.14 ± 0.50	7.18 ± 1.10 *	nd
*Trans*-farnesol	3.94 ± 0.07	4.07 ± 0.38	0.81 ± 0.15
TOTAL	2617.54 ± 27.15	3544.65 ± 66.13	163.16 ± 6.58

nd, not detectable indicates values below the limit of detection (LOD = 0.1 µg·g^−1^).

## Supplementary Materials

Supplementary materials can be found at http://www.mdpi.com/1422-0067/19/9/2805/s1.

## Abbreviations

ACD	Artificial control diet
CAT	Catalase
*CYP71D*/*179*/*180*/*181*/*182*	Cytochrome P450 isoforms
*DXP*	Deoxyxylulose phosphate synthase
*GPPS*	Geranyldiphosphate synthase
GST	Glutathione S-transferase
HIPVs	Herbivore-Induced Plant Volatiles
HW	Herbivore-wounded
SOD	Suoperoxide dismutase
TPS2	γ-terpinene synthase
