# Supplementary material for: Origanum vulgare Terpenoids Induce Oxidative Stress and Reduce the Feeding Activity of Spodoptera littoralis
Source: Int J Mol Sci. 2018 Sep 18;19(9):2805. doi: 10.3390/ijms19092805 (PMC6165561; doi:10.3390/ijms19092805)
Supplement: Supplementary file 1 [file ijms-19-02805-s001.pdf]

Table S1. List of primers used in this work.

Origanum Vulgare Primers		
**Gene**	**Forward Primer (5′→3′)**	**Reverse Primer (5′→3′)**
OvEF1alpha	CTCCAGTTCTTGATTGCCACAC	GCTCCTTTCCAGACCTCCTATC
18S rRNA	ATGATAACTCGACGGATCGC	CTTGGATGTGGTAGCCGTTT
Act	GCTCCAAGGGCTGTGTTCC	TCTTTCTGTCCCATGCCAAC
DXS	CCACCAGGCTTACCCACACAA	GCCACCGCCATCCCTAAAC
TPS2	GTGGCTGAGTTTGGTGGAAGG	TTGGCGTTCTCTAGGTATTCTGC
GPPS	ATGATAAGCGGGCTGCATAG	CCGAAATTCCTCAGCTTCTG
CYP71D178	CAAGGAATGACTGCTGCTGAC	TTGGATTGTGGATTGTTGGAACC
CYP71D179	CGTGGCTTCTCAACCTTCTC	CGCTCTTCTTCACCCTATGC
CYP71D180	GCAAAGAAGAATGCGAGGTC	GATTGAACGTGTCGGGATCT
CYP71D181	TACTGGAAAGACCCCGACAC	CGAACGGGATTAACTCGAAA

Table S2. List of primers used in this work.

Spodoptera Primers		
**Gene**	**Forward Primer (5′→3′)**	**Reverse Primer (5′→3′)**
EF1	CAACCACCCTGGTCAAATCT	ACGGTCGACCTTCTCTTTGA
ACT	GATCTGGCACCACACCTTCT	TCTTCTCCCTGTTGGCCTTA
SOD	GCAGCAGTGTTTGGACACAT	GCACAAACATCAGTCCAGGT
CAT	TCTCCACTGTTGGTGGTGAA	GTTGCCAACAAGATCCCAGT
GST	CAGTAAATTGACGGCCTGGT	CTTCAGTTTGGACTGCACGA

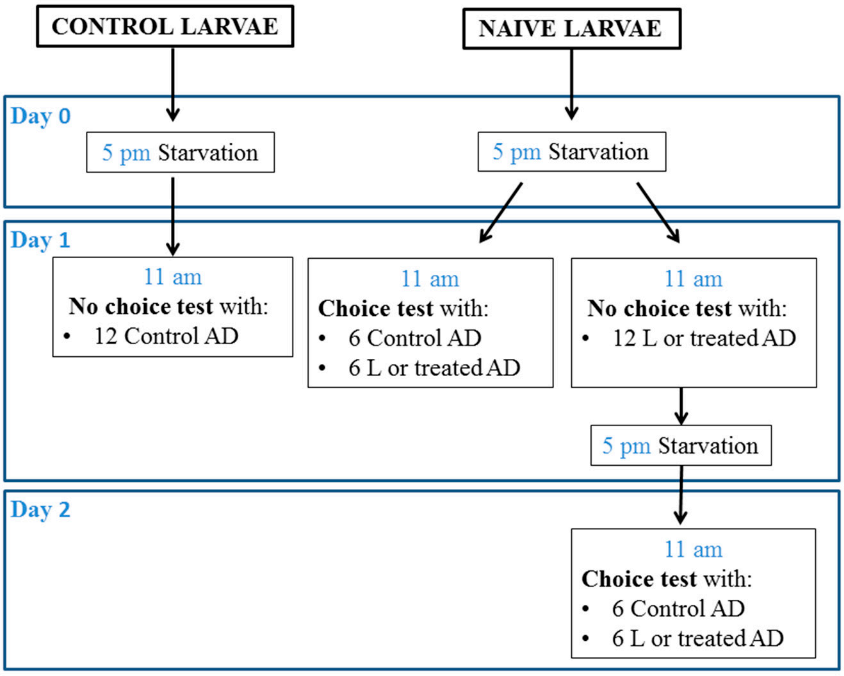

Figure S1. The adopted experimental design for choice tests. AD = artificial diet; L = oregano leaves; treated AD = artificial diet supplemented with carvacrol, ϱ-cymene or γ-terpinene).
